# Use of a temporary Ilizarov rod and cement spacer in the treatment of long bone infection

**DOI:** 10.1308/rcsann.2014.96.1.77

**Published:** 2014-01

**Authors:** A Vaughan, J Mutimer, S Clint

**Affiliations:** Gloucestershire Hospitals NHS Foundation Trust,UK

## BACKGROUND

**Figure 1:**
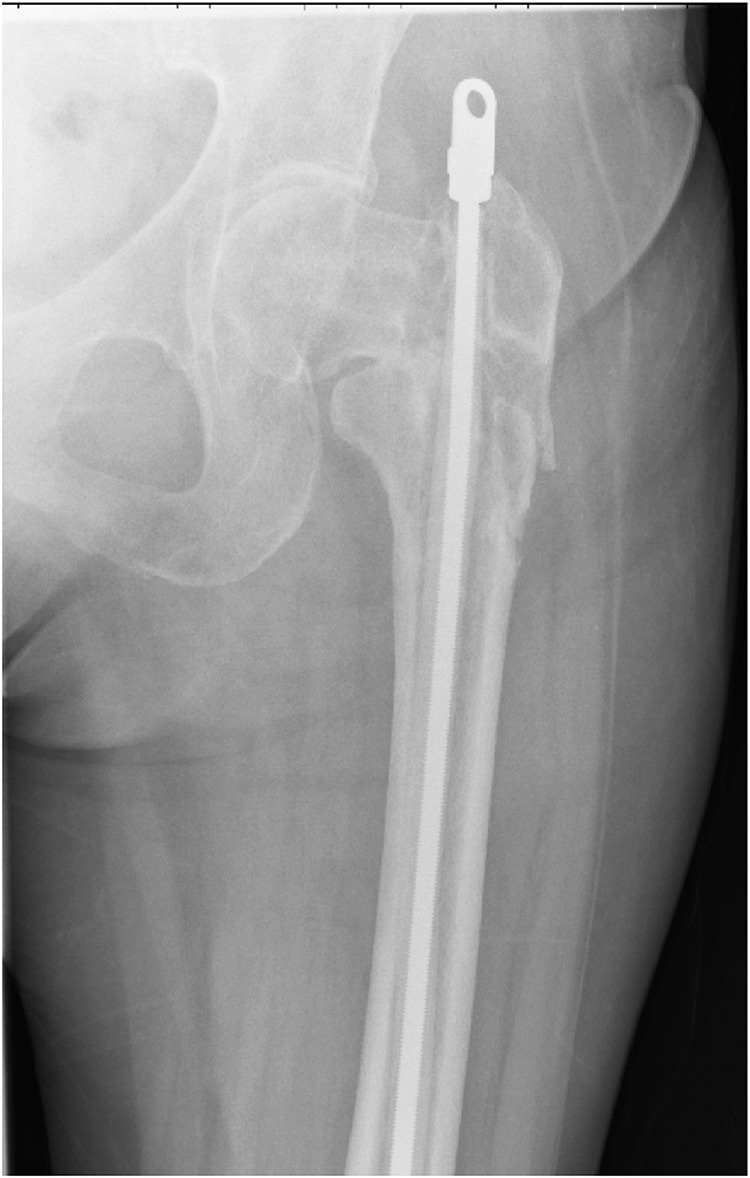
X-ray showing Ilizarov rod with cement used as temporary spacer following removal of infected left femoral intramedullary nail

The treatment of an infected intramedullary nail is a challenging and serious complication. Normally, treatment involves the removal of metalwork, followed by washout and debridement of the infected bone. This can leave the bone and any fracture unsupported. We promote using a threaded Ilizarov rod encased with antibiotic impregnated Palacos® cement (Heraeus, Wehrheim, Germany) to act as a temporary spacer when treating intramedullary infection (Fig 1).

## TECHNIQUE

The medullary canal is reamed and irrigated. An Ilizarov threaded rod is chosen, appropriate to the canal length of the affected bone. A chest tube of similar internal diameter to the reamed canal is selected. While still liquid, the tube is filled with cement and the Ilizarov rod is immediately inserted into it. Once set, the chest tube can be peeled off using a surgical knife. A female hinge, secured with a nut, can be placed on the proximal end, acting as a hook. The cement and rod construct is then inserted down the medullary canal.

## DISCUSSION

**Figure 2:**
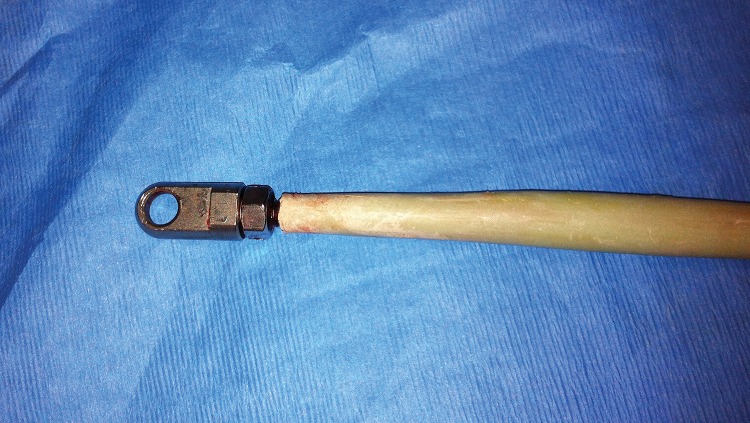
Appearance of hook with cemented Ilizarov rod following removal

This technique allows local delivery of high concentration antibiotics to the canal in its entirety. The hook at the end of the rod facilitates easy removal, and the threads provide stable integration of cement and rod so no cement is left behind on removal (Fig 2). The use of a tube as a mould provides a smooth spacer of uniform thickness, aiding insertion. The rod acts as a temporary spacer, providing mechanical support to an infected non-union, and reduces the risk of pathological fracture in an intact bone.

